# Material Basis of Chinese Herbal Formulas Explored by Combining Pharmacokinetics with Network Pharmacology

**DOI:** 10.1371/journal.pone.0057414

**Published:** 2013-02-28

**Authors:** Lixia Pei, Yuanwu Bao, Sheng Liu, Jin Zheng, Xiuping Chen

**Affiliations:** 1 Pharmacology Laboratory of Traditional Chinese Medicine, Longhua Hospital, Shanghai University of Traditional Chinese Medicine, Shanghai, China; 2 State Key Laboratory of Quality Research in Chinese Medicine, Institute of Chinese Medical Sciences, University of Macau, Macao, China; Cleveland Clinic Lerner Research Institute, United States of America

## Abstract

The clinical application of Traditional Chinese medicine (TCM), using several herbs in combination (called formulas), has a history of more than one thousand years. However, the bioactive compounds that account for their therapeutic effects remain unclear. We hypothesized that the material basis of a formula are those compounds with a high content in the decoction that are maintained at a certain level in the system circulation. Network pharmacology provides new methodological insights for complicated system studies. In this study, we propose combining pharmacokinetic (PK) analysis with network pharmacology to explore the material basis of TCM formulas as exemplified by the Bushen Zhuanggu formula (BZ) composed of *Psoralea corylifolia* L., *Aconitum carmichaeli* Debx., and *Cnidium monnieri* (L.) Cuss. A sensitive and credible liquid chromatography tandem mass spectrometry (LC-MS/MS) method was established for the simultaneous determination of 15 compounds present in the three herbs. The concentrations of these compounds in the BZ decoction and in rat plasma after oral BZ administration were determined. Up to 12 compounds were detected in the BZ decoction, but only 5 could be analyzed using PK parameters. Combined PK results, network pharmacology analysis revealed that 4 compounds might serve as the material basis for BZ. We concluded that a sensitive, reliable, and suitable LC-MS/MS method for both the composition and pharmacokinetic study of BZ has been established. The combination of PK with network pharmacology might be a potent method for exploring the material basis of TCM formulas.

## Introduction

Traditional Chinese medicine (TCM), the ancient medicine popular in China and surrounding areas, has been recognized as a representative of complementary and alternative medicine. Though with a long period of clinical practice, its effectiveness and beneficial contribution to public health and disease control has not been fully established [1], [2]. From basic research point of view, although there has been considerable volume of research into TCM in recent years, the quality of the evidence and the research strategy are generally very poor. The most common practice in TCM is the use of herbal combinations called formulas, which consists of several herbs. With the resurgence of enthusiasm for drug research and development based on natural products, the proposed multitarget drug discovery strategy, and the implementation of TCM modernization plan in China, more attention has been paid to TCM [3]–[5]. However, the clarification of the material basis of TCM formulas is the fundamental prerequisite for its worldwide recognition and acceptance.

Though TCM formulas contain several or even dozens of herbs and definitely involve a variety of compounds, the number of therapeutic ones should be greatly decreased because of poor absorption, low bioavailability, low content in the raw herbs, and so on. Furthermore, the low clinical dosage (commonly 6 g to 9 g for most Chinese herbs as approved by the Chinese pharmacopoeia) excludes some effective compounds with extremely low content. In addition, the decoction, the traditional routine of formula preparation, ignored by many researchers in this area, also excludes some poorly water-soluble components. According to the basic concentration–response concept in classical pharmacology, we hypothesize that the material basis for TCM formulas are the total absorbable bioactive compounds that reach certain concentrations in circulatory system [6]. Thus, for a specific TCM formula, the material basis might be very small and limited.

Pharmacokinetics (PK) investigates the action of drugs in the body over a period of time, which mainly includes absorption, distribution, metabolism, and excretion. PK has played an important role in drug research and development [7]–[9]. The combination of PK with pharmacodynamics (PD) (PK/PD model) has been introduced in drug evaluation for decades [10]. PK and PD integration provides a powerful means of enhancing our understanding of the dose–response [11] and has been proposed as an integrated approach to drug development [12]. Furthermore, with the development of metabolomics, an integrated metabolomics and PK strategy may be a choice for multicomponent drug evaluation, especially for herbal medicines [13]. However, PK studies on TCM formulas are a big challenge with many interesting hot points and difficulties [14]. Though several attempts have been made to investigate the PK profile of some compounds in TCM formulas, such as Xiexin decoction [15] and Liu-Wei-Di-Huang-Wan [16], and to screen the components of Yin-Chen-Hao-Tang absorbed in rat plasma [17], the potential significance of PK to TCM formulas remains unexplored.

Network view, strategy, and analysis for understanding the complicated systems of social science [18], biology [19], and medicine [20] has been developed fast in recent years. Network pharmacology was firstly introduced by Hopkins in 2008 originally as an approach to drug design that encompasses systems biology, network analysis, connectivity, redundancy, and pleiotropy [21]. Recently, the concept of network has been applied to TCM for screening synergistic drug combinations [22], establishing a workflow for network-based TCM pharmacology [23], uncovering rules for combination [24], and predicting drug targets [25].

Based on this assumption, we propose a strategy to explore the material basis of TCM formulas by integrating PK with network pharmacology, which includes four steps. First, a reliable and highly sensitive analytical method for the simultaneous determination of multiple components within a formula should be established. Second, the multiple components in formula/decoctions should be quantitatively assayed. Third, the plasma concentrations should be determined after formula administration and PK profile should be calculated to screen for candidates. Fourth, network analysis and confirmation should be performed. Fulfilling this idea is a major challenge because the methods for PK are required to be (1) highly sensitive, because most constituents in TCM have low concentrations, especially after oral administration and (2) highly specific, because many of the constituents in TCM are structurally similar and chemically isomeric. Furthermore, only a limited sample amount is available for *in vivo* PK studies. Fortunately, the development of analytical technology, particularly liquid chromatography tandem mass spectrometry (LC-MS/MS) has provided a useful tool.

The Bushen Zhuanggu formula (BZ), composed of *Psoralea corylifolia* L., *Aconitum carmichaeli* Debx., and *Cnidium monnieri* (L.) Cuss, has been clinically prescribed in our hospital for many years as an alternative therapy for metastatic breast cancer treatment. A clinical retrospective cohort study showed that BZ acts as an adjuvant in breast cancer patients with bone metastasis by improving bone pain, reducing the incidence of bone-related events, and decreasing and delaying osteolytic lesions. Our recent studies also demonstrated that *P. corylifolia* and *C. monnieri* inhibit the bone metastasis of breast cancer *in vivo*, possibly by affecting the OPG/RANKL/RANK system [26], [27]. The main components identified from these herbs are psoralen, isopsoralen, corylifolin, corylifolinin, psoralidin, and so on, from *P. corylifolia* [28]–[30]; aconitine, hypaconitine, mesaconitine, benzoylaconine, benzoylhypaconine, benzoylmesaconitine, and so on, from *A. carmichaelii* [31]–[33]; and osthole, bergapten, imperatorin, xanthotoxin, from *C. monnieri* [34], [35].

In the present study, we established a LC-MS/MS method to quantify 15 compounds simultaneously in BZ decoction and rat plasma. Furthermore, the PK profiles of 5 compounds after BZ administration in rats were calculated. In addition, network analysis of the five compounds was also performed.

## Materials and Methods

### Materials

**Figure 1:**
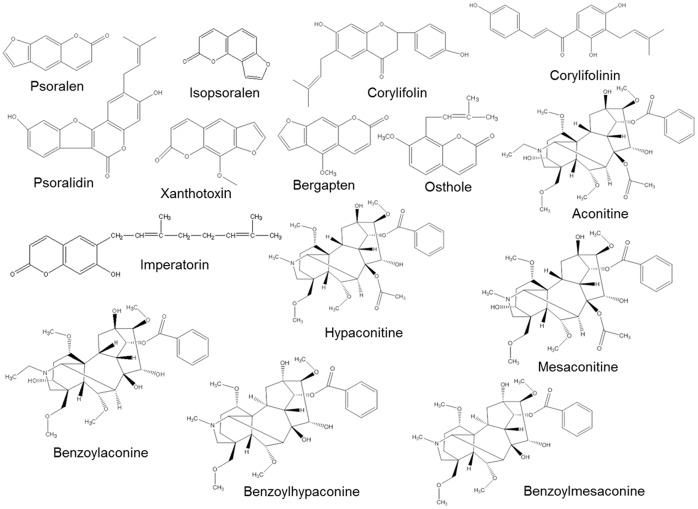
The chemical structures of 15 constituents in BZ.

Fifteen standard compounds (psoralen, isopsoralen, corylifolin, corylifolinin, psoralidin, xanthotoxin, bergapten, osthole, imperatorin, aconitine, hypaconitine, mesaconitine, benzoylaconine, benzoylhypaconine, and benzoylmesaconitine; Figure 1), were purchased from Weikeqi Biological Technology Co., Ltd. (Chengdu, China). The purities of the compounds were >98% as determined by high-performance liquid chromatography (HPLC). The stock solution of each standard was prepared at a concentration of 1 mg/mL in 50% MeOH and stored at −80°C until use. The herbs *P. corylifolia*, *A. carmichaeli*, and *C. monnieri* were purchased from Longhua Hospital (Shanghai, China). HPLC-grade methanol (MeOH, 99.9%) and formic acid were obtained from Fisher (Fair Lawn, NJ, USA). Distilled water was produced from a Millipore water purification system.

### Preparation of Formula Extracts

*P. corylifolia* (180 g), *C. monnieri* (54 g), and *A. carmichaeli* (36 g), (consistent with clinical prescription ratio) were mixed together and soaked with 2,160 mL water (1∶8, g/v) at 25°C for 30 min, followed by boiling for 1 h. The supernatant was collected after centrifugation at 4000×*g* for 10 min. The residue was extracted again with 1620 mL water (1∶6, g/v). The two supernates were pooled and concentrated to a total volume of 270 mL (D7) for PK studies.

The effects of herbal interactions on the solubility of the constituents were determined by preparing another six decoctions following the same procedure for D7∶6 g of *A. carmichaeli* (D1); 30 g of *P. corylifolia* (D2); 9 g of *C. monnieri* (D3); 30 g of *P. corylifolia* and 6 g of *A. carmichaeli* (D4); 6 g of *A. carmichaeli* and 9 g of *C. monnieri* (D5); and 30 g of *P. corylifolia* and 9 g of *C. monnieri* (D6). All decoctions were diluted 100-fold and 1000-fold with distilled water before injection for LC-MS/MS analysis.

### Animal Study

Male Sprague–Dawley rats (∼250 g) purchased from Sino-British Sippr/BK Lab Animal, Ltd. (Shanghai, China) were housed in air-conditioned animal quarters with alternating 12 h light/dark cycles at a room temperature of 22°C±2°C and a relative humidity of 50%±10%. Commercial rat chow and water were given *ad libitum*. The animal studies were approved by the Review Committee of Animal Care and Use at Longhua Hospital. The rats were fasted overnight (∼12 h) and had free access to water throughout the experimental period. Five rats were given a single oral dose of D7 at 4 mL/kg, which was designed based on the clinical dosage for human. Blood samples (∼150 µL) were collected *via* a jugular vein catheter from non-restrained, non-sedated animals into heparinized tubes before and at 0.083, 0.25, 0.5, 1, 2, 3, 4, 6, 8, 10, and 24 h after administration. After centrifugation at 4000×*g* for 10 min at 4°C, duplicates of 25 µL of the plasma samples were obtained and were immediately stored at −80°C until analysis. After the experiments, the rats were sacrificed with ether anesthesia.

### Plasma Sample Preparation

For protein precipitation, 75 µL of MeOH were added to 25 µL of plasma samples. After vortexing for 2 min and centrifugation for 5 min at 13000×*g* at 4°C, 80 µL aliquots of the supernatant liquid were transferred into autosampler vials for LC-MS/MS analysis.

### LC-MS/MS Analysis

The LC-MS/MS system consisted of an Agilent HPLC 1200 series system and a CTC autosampler coupled with an API 4000 triple-quadrupole mass spectrometer equipped with a TurboIonSpray ion source. The LC-MS/MS system was controlled using the Analyst 1.4.2 software, which also acquired and processed the data.

LC separation was performed on a 5 µm Agel Luna C_18_ (50 mm×2.1 mm, i.d.) at room temperature with a 2 µm filter used before the analytical column. The LC mobile phase was H_2_O containing 0.1% formic acid (A) and MeOH (B) at a flow rate of 0.30 mL/min. A gradient elution program was used as following: 0→2.5 min, B% 60→90; 2.5→2.6 min, B% 90→60; and 2.6→4 min, B% 60→60. The injection volume was 10 µL and the temperature of the autosampler was set at 4°C to keep the samples stable during the analysis.

The tuning solutions (10 µg/mL) of the individual analytes were prepared by diluting the corresponding primary stock solutions with 50% MeOH. The tuning solutions were delivered at 5.0 µL/min using a syringe pump and combined through a PEEK tee-connector with the mobile phase delivered at 0.3 mL/min with the LC pump. The instrumental parameters of the MS spectrometers in positive ion ESI mode were optimized to achieve maximum ionization of the analyte molecules and the generation of characteristic fragment ions. The precursor-to-product ion pairs used for the multiple reaction monitoring (MRM) were 187→131 (*m/z*) for psoralen and isopsoralen; 325→149 (*m/z*) for corylifolin and corylifolinin; 337→281 (*m/z*) for psoralidin; 216→202 (*m/z*) for xanthotoxin and bergapten; 244→131 (*m/z*) for osthole; 271→203 (*m/z*) for imperatorin; 646→586 (*m/z*) for aconitine; 617→557 (*m/z*) for hypaconitine; 632→572 (*m/z*) for mesaconitine; 604→554 (*m/z*) for benzoylaconine; 574→542 (*m/z*) for benzoylhypaconine; and 590→540 (*m/z*) for benzoylmesaconitine. The scan time for each pair was set to 50 ms. The operating parameters of the mass spectrometer included compound-dependent and source-dependent considerations. The optimized source-dependent parameters consisted of the flow rates of curtain gas, gas 1, gas 2, collision gas (CAD), capillary temperature (TEM), and ion spray voltage. The compound-dependent parameters for the test compounds included the declustering potential (DP), entrance potential (EP), collision energy (CE), and collision cell exit potential (CXP).

### Calibration Curve Construction and Partial Validation

The primary stock solutions were pooled and mixed to obtain an intermediate stock solution at 50 µg/mL in 50% MeOH for each compound. The intermediate stock solution was then serially diluted with 50% MeOH to get the working solutions at 10,000, 7,500, 5,000, 1,000, 500, 100, 50, 25, and 10 ng/mL.

Standard curves were constructed by preparing calibrator pool solutions containing 15 compounds from the serial working solution by dilution with water (1∶9, v/v) to 1,000, 500, 100, 50, 10, 5, 2.5, and 1 ng/mL. Calibration graphs were constructed using the linear regression of each compound peak area to its nominal concentration (X, ng/mL) by weighing the reciprocal concentration (1/X^2^). The intra-batch accuracy and precision of the analytical method described here was determined by analyzing water solutions containing 15 compounds at 4 different nominal concentrations (2.5, 5, 50, and 750 ng/mL). The quality control values were calculated from the regression equations.

Standard curves, intra-batch accuracy, and precision for the quantification of the 15 compounds in the rat plasma samples were constructed similarly. The accuracy was measured as the difference between the nominal value and the measured value, expressed as a percentage of the nominal value. Precision was expressed as the coefficient of variation (CV), i.e., the standard deviation divided by the mean multiplied by 100.

The assay was partially validated against specificity, recovery, and matrix effect. The specificity of the assay was assessed by analyzing six different blank rat plasma samples to exclude the interference of endogenous plasma components.

The recovery of each compound was determined by comparing the peak area of the precipitated plasma samples at three QC concentrations in three replicates with those of the neat standard in post- precipitated samples. The matrix effect was determined at the same three concentrations by comparing peak areas of neat standard in post-precipitated samples (from six batches of rat plasma) with those of other neat solution (water) standards. The recovery rates and matrix effects were determined at 2.5, 5, 50, and 750 ng/mL.

### Pharmacokinetic Analysis

The plasma concentration–time data for D7 were analyzed with the WinNonlin 5.2.1 software (Pharsight, Mountain View, CA, USA) using a non-compartmental model. The peak plasma concentration (*C*_max_) and the corresponding time (*T*_max_) were directly obtained from the concentration data.

### Data Preparation and Mining

Enough data were obtained for the primary screening and further establishment of the network for potential candidates by retrieving several biological and drug discovery-related informative databases including NCBI’s Entrez Gene, NCBI’s PubMed, and CNKI. The data were obtained before May 18, 2012.

### Network Analysis

Cytoscape has been widely used for the reconstruction and visualization of networks. InterologFinder is designed to retrieve protein–protein interactions (PPIs) from both known and predicted PPI data sets (http://interologfinder.org/interologFiles/aboutus.html). In this study, we constructed two networks using Cytoscape 2.8.2 namely, the drug–herb interaction network (D–H network) for herb combination and TCM formula and the drug–target association network (D–T network). The D–H network was constructed by linking the compounds when their interaction was reported or confirmed in the present study, both *in vitro* and *in vivo*. The D–T networks were established by linking the compounds and their potential targets and associated proteins and genes.

## Results

### LC-MS/MS Quantification Method

**Figure 2:**
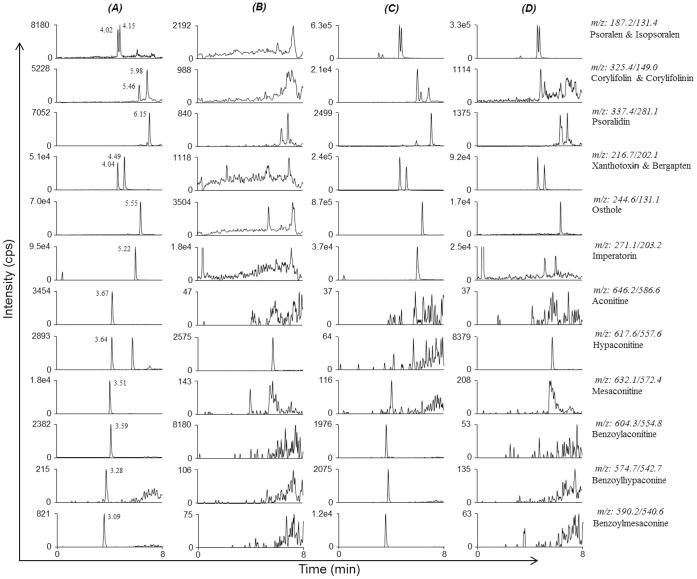
LC-MS/MS chromatograms of 15 constituents (A) blank rat plasma sample spiked with 15 standards; (B) blank rat plasma sample; (C) decoction of BZ; (D) rat plasma sample collected after single oral dose of BZ.

Under our conditions, a sensitive and reliable analytical method for the simultaneous quantification of 15 compounds has been developed. Representative chromatograms of the compounds in pure solvent (water), formula extract, and rat plasma extract are shown in Fig. 2, which indicates that the developed method is selective for all 15 compounds tested in the formula decoction and rat plasma without any obvious interference.

The regression equations for the 15 compounds showed good linearity between concentration and ESI-MS/MS response over the dynamic range from 1 or 5 to 100 or 1,000 ng/mL in both the matrices of pure water and rat plasma (Table 1). The recovery rates for all 15 compounds were consistent at 2.5, 5, 50, and 700 ng/mL and ranged from 83% to 107%. The endogenous components in rat plasma inhibited the mass spectrometry response. However, the inhibitory effect was similar for each compound at 2.5, 5, 50, or 700 ng/mL and ranged from 69% to 81%.

**Table 1:** LC-MS/MS method validation for 15 constituents from BZ.

Constituent	Water-based				Rat plasma-based			
	Regression equation (Dynamic range, *r*)	QC1 2 or 5 ng/mL (Acc: %; Pre: %)	QC2 5 or 50 ng/mL (Acc: %; Pre: %)	QC3 50 or 750 ng/mL (Acc: %; Pre: %)	Regression equation (Dynamic range, *r*)	QC1 2 or 5 ng/mL (Acc: %; Pre: %)	QC2 5 or 50 ng/mL (Acc: %; Pre: %)	QC3 50 or 750 ng/mL (Acc: %; Pre: %)
Psoralen	Y = 5830X +3920 (1–1000 ng/mL, 0.998)	1.91±0.15 (96%; 7.8%)	44.7±2.7 (89%; 6.0%)	680.5±40.7 (91%; 6.0%)	Y = 2910X –88.4 (1–1000 ng/mL, 0.996)	1.85±0.20 (93%; 710.8%)	53.4±4.4 (107%; 8.2%)	810.4±33.9 (108%; 4.2%)
Isopsoralen	Y = 3940X +3090 (1–1000 ng/mL, 0.991)	1.98±0.10 (99%; 5.0%)	53.3±4.5 (107%; 8.4%)	735.2±28.7 (98%; 3.9%)	Y = 2270X –616 (1–1000 ng/mL, 0.995)	1.73±0.15 (87%; 8.7%)	48.7±3.9 (97%; 8.0%)	690.7±56.1 (92%; 8.1%)
Corylifolin	Y = 2140X –52.6 (1–1000 ng/mL, 0.996)	1.87±0.19 (94%; 10.2%)	48.3±1.5 (97%; 3.1%)	834.7±70.5 (111%; 8.4%)	Y = 602X –98 (1–1000 ng/mL, 0.992)	1.93±0.22 (97%; 11.4%)	44.9±3.3 (90%; 7.3%)	770.4±65.9 (103%; 8.6%)
Corylifolinin	Y = 2400X –145 (1–1000 ng/mL, 0.990)	1.78±0.20 (89%; 11.2%)	45.9±5.1 (92%; 10.2%)	770.9±65.9 (103%; 8.5%)	Y = 1430X +7080 (5–1000 ng/mL, 0.995)	4.37±0.50 (87%; 11.4%)	51.7±4.1 (103%; 7.9%)	789.3±43.5 (105%; 5.5%)
Psoralidin	Y = 2150X +1140 (1–1000 ng/mL, 0.992)	1.93±0.21 (97%; 10.9%)	52.4±2.5 (105%; 4.7%)	745.9±33.8 (100%; 4.5%)	Y = 650X –307 (1–1000 ng/mL, 0.993)	2.12±0.23 (106%; 10.8%)	53.7±6.3 (107%; 11.7%)	710.2±80.5 (95%; 11.3%)
Xanthotoxin	Y = 21400X +708 (1–100 ng/mL, 0.999)	1.72±0.15 (86%; 8.7%)	4.93±0.52 (99%; 10.5%)	47.9±5.1 (96%; 10.6%)	Y = 20300X –271 (1–100 ng/mL, 0.997)	2.21±0.13 (111%; 5.9%)	5.18±0.18 (104%; 3.5%)	44.9±5.8 (90%; 12.9%)
Bergapten	Y = 20100X +3910 (1–100 ng/mL, 0.998)	1.88±0.12 (94%; 6.4%)	4.48±0.24 (90%; 5.3%)	51.5±1.5 (103%; 2.9%)	Y = 15600X –1650 (1–100 ng/mL, 0.998)	1.98±0.17 (99%; 8.6%)	4.96±0.57 (99%; 11.5%)	49.5±3.2 (99%; 6.5%)
Osthole	Y = 22500X +5040 (1–100 ng/mL, 0.998)	1.74±0.23 (87%; 13.2%)	5.20±0.48 (104%; 9.2%)	48.9±3.2 (98%; 6.5%)	Y = 25900X +3830 (1–100 ng/mL, 0.997)	1.79±0.19 (90%; 10.6%)	4.75±0.28 (95%; 5.9%)	54.1±1.1 (108%; 2.0%)
Imperatorin	Y = 47300X +10300 (1–100 ng/mL, 0.995)	1.93±0.14 (97%; 7.3%)	4.87±0.33 (97%; 6.7%)	55.1±6.3 (110%; 11.4%)	Y = 32400X +2720 (1–100 ng/mL, 0.996)	2.10±0.25 (105%; 11.9%)	5.54±0.19 (111%; 3.4%)	47.1±3.2 (94%; 6.8%)
Aconitine		1.81±0.17 (91%; 9.4%)	49.2±5.5 (98%; 11.1%)	783.9±64.7 (105%; 8.3%)	Y = 1480X +265 (1–1000 ng/mL, 0.993)	1.96±0.21 (98%; 10.7%)	48.3±4.4 (97%; 9.1%)	689.5±20.1 (92%; 2.9%)
Hypaconitine		1.71±0.07 (86%; 5.0%)	53.7±4.8 (107%; 8.9%)	738.1±54.5 (98%; 7.4%)	Y = 1260X –72.8 (1–1000 ng/mL, 0.995)	2.04±0.05 (102%; 2.5%)	53.9±2.1 (108%; 3.9%)	793.1±25.0 (106%; 3.2%)
Mesaconitine		1.94±0.11 (97%; 5.7%)	52.4±1.9 (105%; 3.6%)	647.8±35.0 (86%; 5.4%)	Y = 6520X +518 (1–1000 ng/mL, 0.994)	2.13±0.09 (107%; 4.2%)	44.1±4.8 (88%; 10.9%)	735.5±70.1 (98%; 9.5%)
Benzoylaconitine		2.10±0.23 (105%; 11.0%)	52.9±3.8 (106%; 7.2%)	723.9±10.5 (97%; 1.5%)	Y = 1020X –270 (1–1000 ng/mL, 0.991)	1.89±0.13 (95%; 6.9%)	46.9±1.5 (94%; 3.2%)	770.1±15.3 (103%; 2.0%)
Benzoylmesaconine		2.14±0.15 (107%; 7.0%)	47.3±5.1 (89%; 10.8%)	707.6±80.1 (94%; 11.3%)	Y = 419X –49.3 (1–1000 ng/mL, 0.998)	1.82±0.16 (91%; 78.8%)	55.2±3.1 (110%; 5.6%)	745.1±22.5 (99%; 3.0%)
Benzoylhypacoitine		1.96±0.23 (98%; 11.7%)	50.4±1.3 (100%; 2.6%)	688.6±20.5 (92%; 3.0%)	Y = 13.1X –7.91 (5–1000 ng/mL, 0.994)	4.43±0.20 (89%; 4.5%)	45.2±3.3 (90%; 7.3%)	738.1±44.9 (98%; 6.1%)

### Chemical Analysis of BZ

Chemical analysis of BZ revealed that only 3 compounds (aconitine, hypaconitine, and mesaconitine) were not found in D7, given that their concentrations were below the LLOQ of the analytical method (Table 2). Among the remaining 12 compounds, only psoralidin was below 10 µg/mL.

**Table 2:** Contents of 15 constituents in 7 decoctions.

Herb	Constituent	Content in Decoction (µg/mL)						
		D1	D2	D3	D4	D5	D6	D7
*Psoralea corylifolia L*	Psoralen	ND	165.40 (100)	ND	148.40 (90)	ND	133.40 (81)	157.80 (95)
	Isopsoralen	ND	260.20 (100)	ND	250.80 (96)	ND	216.20 (83)	242.80 (93)
	Corylifolin	ND	65.40 (100)	ND	53.80 (82)	ND	36.56 (56)	51.02 (78)
	Corylifolinin	ND	25.64 (100)	ND	23.96 (93)	ND	14.52 (57)	27.08 (106)
	Psoralidin	ND	2.47 (100)	ND	2.67 (108)	ND	1.48 (60)	2.78 (113)
	**Subtotal**	**ND**	**519.11 (100)**	**ND**	**479.64 (92)**	**ND**	**402.16 (77)**	**481.46 (93)**
*Cniclium monnieri (L.) Cuss*	Osthole	ND	ND	122.00 (100)	ND	130.21 (107)	71.43 (59)	93.00 (76)
	Bergapten	ND	ND	51.20 (100)	ND	50.42 (98)	22.83 (45)	25.44 (50)
	Xanthotoxin	ND	ND	33.68 (100)	ND	33.16 (98)	12.64 (38)	14.96 (44)
	Imperatorin	ND	ND	30.78 (100)	ND	29.36 (95)	18.56 (60)	27.92 (91)
	**Subtotal**	**ND**	**ND**	**237.66 (100)**	**ND**	**243.12 (102)**	**125.41 (53)**	**161.32 (68)**
*Aconitum carmichaeli Debx*	Benzoylaconitine	1.85 (100)	ND	ND	1.84 (99)	1.65 (89)	ND	1.74 (94)
	Benzoylmesaconine	165.89 (100)	ND	ND	170.65 (103)	180.34 (109)	ND	150.33 (91)
	Benzoylhypacoitine	149.57 (100)	ND	ND	160.21 (107)	135.83 (91)	ND	158.33 (106)
	**Subtotal**	**317.31 (100)**	**ND**	**ND**	**332.7 (105)**	**317.82 (100)**	**ND**	**310.40 (98)**

The chemical compositions of D1–D6 clearly showed significant interactions among the solubility of the herb constituents (Table 2). Compared with single herb solutions, *C. monnieri* and *P. corylifolia* dramatically decreased each other’s content by 56%–83% and 38%–60%, respectively, whereas the influences between *A. carmichaeli* and *P. corylifolia* or *C. monnieri* were negligible. Interestingly, when the 3 herbs were mixed together, the extract efficiencies increased to some degree. In D7, the constituents of *P. corylifolia* were similar to those of D1, except for corylifolin (78%). More constituents were extracted from *C. monnieri* compared with D5, which ranged from 44% to 91% of those in single herb solutions. However, the constituents from *A. carmichaeli* remained unchanged in these decoctions.

### PK Profile of BZ

**Figure 3:**
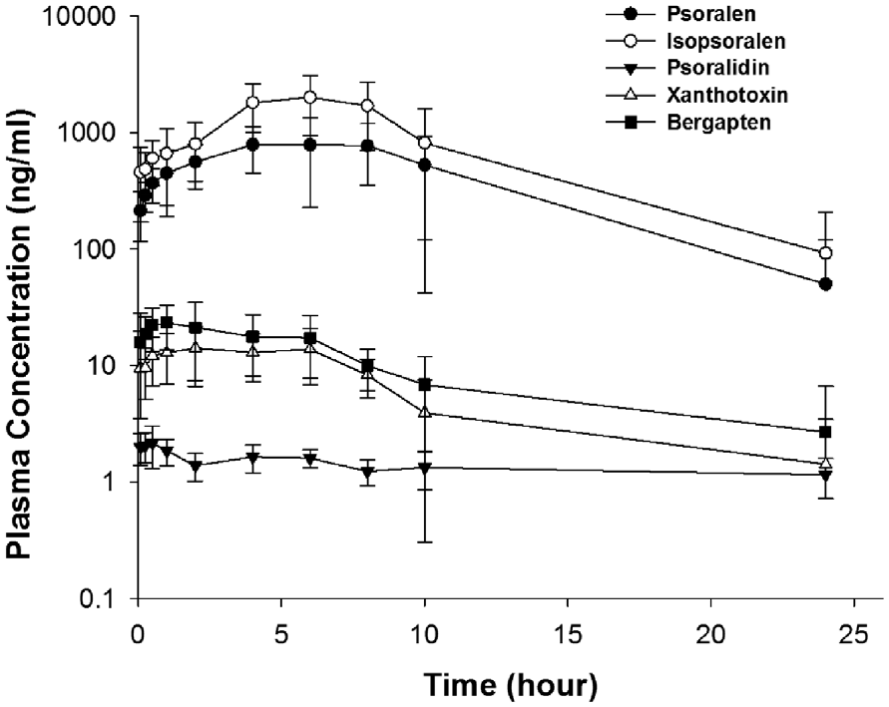
Plasma concentration-time curves for psoralen, isopsoralen, psoralidin, xanthotoxin, and bergapten in SD rats after single oral administration of BZ.

Although 12 compounds were detected in D7, 4 (aconitine, hypaconitine, mesaconitine, and benzoylaconitine) were not detected in rat plasma after D7 administration. Of the other 8 compounds detected, only psoralen (157.80 µg/rat), isopsoralen (242.80 µg/rat), xanthotoxin (14.96 µg/rat), bergapten (25.44 µg/rat), and psoralidin (2.78 µg/rat) were measureable in rat plasma up to 10 h or 24 h after a single oral dosage. These compounds showed quite different plasma concentration–time curves (Figure 3). The *AUC_0–24 h_* for psoralen and isopsoralen (9977±5955 ng•h/mL and 20350±11537 ng•h/mL) were much higher than those of xanthotoxin and bergapten (162±64.7 and 143±73.2 ng•h/mL). Psoralidin was measurable in plasma, but the concentration was maintained at a relatively low level up to 24 h post-dose. The PK parameters are shown in Table 3. Psoralen, isopsoralen, xanthotoxin, and bergapten have comparable elimination half-lives (*T_1/2_*), about 3 h to 4 h, whereas psoralidin has a much longer *T_1/2_* of about 24 h.

**Table 3:** Pharmacokinetic parameters of 5 constituents after single oral administration of BZ in SD rats.

PK parameter	Constituent				
	Psoralen	Isopsoralen	Psoralidin	Bergapten	Xanthotoxin
*T_max_* (h)	6	6	0.5	1	1
*C_max_* (ng/mL)	929±431	2237±824	2.57±0.53	20.5±7.22	31.7±9.55
*T_1/2_* (h)	3.18±0.24	3.53±1.03	23.7±1.7	4.14±0.70	4.56±0.71
*AUC_last_* (h·ng/mL)	9977±5955	20350±11537	31.90±3.32	143±73.2	162±64.7
*AUC_all_* (h·ng/mL)	10193±6005	20959±12119	64.51±8.46	158±77.9	209±89.5
*CL/F* (mL/h/kg)	83.5±45.4	65.8±42.9	173.0±21.0	484±228	576±246
*V_z_/F* (mL/kg)	384±227	308±181	5873±395	2930±1570	3694±1522
*AUMC_last_* (h·h·ng/mL)	69918±49475	150025±99963	329±44	924±716	667±270
*AUMC_all_* (h·h·ng/mL)	74305±51503	168803±119110	2240±462	1270±958	1445±719
*MRT* (h)	6.71±0.79	7.13±0.66	10.3±0.39	5.65±2.16	4.06±0.41

### D–H Network Construction

**Figure 4:**
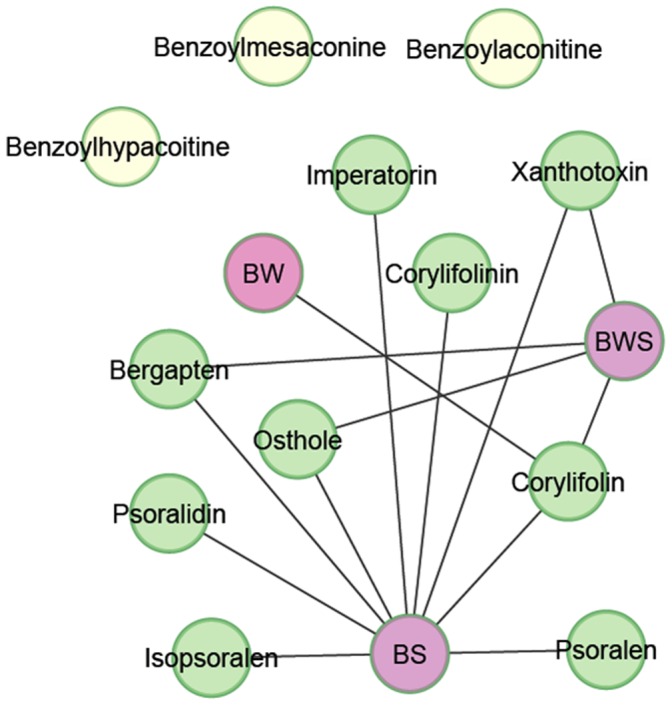
The drug–herb interaction network (D–H network) for BZ. BS, *P. corylifolia* and *C. monnieri*; BW, *P. corylifolia* and *A. carmichaelii*; BWS, *P. corylifolia*, *C. monnieri* and *A. carmichaelii*.

The D–H network was constructed using Cytoscape version 2.8.2, as indicated in Figure 4, which clearly demonstrates that most of the compounds in BZ are affected by the compatibility of *P. corylifolia* and *C. monnieri*. The three main compounds from *A. carmichaeli* were outside the network. Among the 12 compounds detected in the decoctions, corylifolin is the most likely to be affected.

### D–T Network Construction

**Figure 5:**
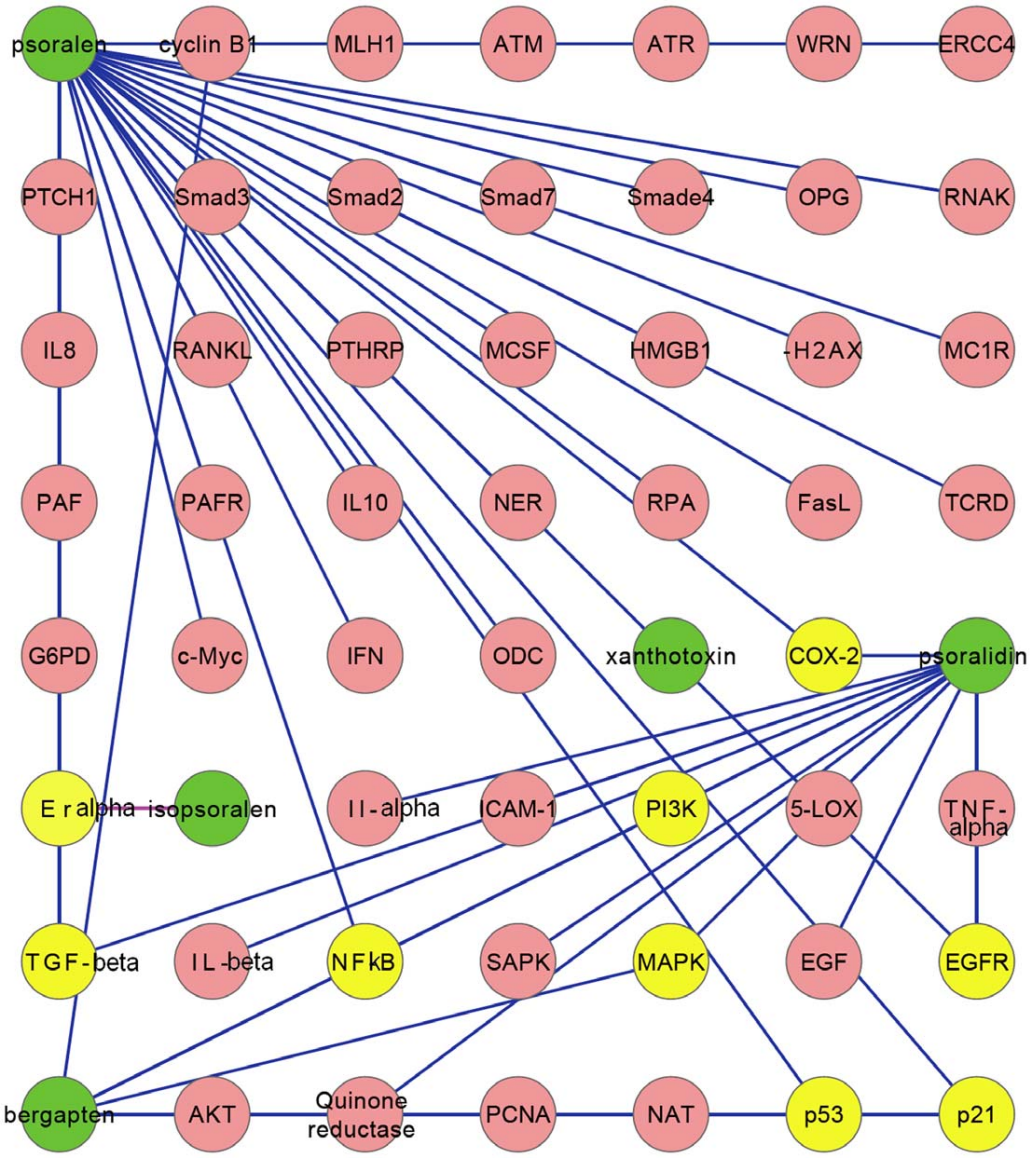
The drug–target association network (D–T network) for BZ. Green, the five compounds from BZ; Pink, the target-associated genes; Yellow, the shared genes among the five compounds.

The PK profiles showed that only 5 out of 15 compounds reached detectable concentrations in rat plasma, as indicated by LC-MS/MS. The potential targets and the associated genes and proteins for the five compounds were obtained by searching NCBI Entrez Gene, NCBI PubMed, CNKI, and so on. A total of 37 results for psoralen, 14 results for psoralidin, 9 results for bergapten, and 1 result for isopsoralen were obtained. No item was related to xanthotoxin. Cytoscape version 2.8.2 was used to model the drug-target–associated protein-signaling network (Figure 5). Only xanthotoxin was not involved in the network. Among these genes and proteins, nine (yellow) were shared by these compounds.

## Discussion

The identification of the material basis of TCM formulas has been a major challenge worldwide. Based on classic pharmacologic principles and the characteristics of TCM prescription, we considered that the number of material basis might be overestimated in conventional *in vitro* assays. Both *in vitro* and *in vivo* data were considered by combining PK with network pharmacology firstly in this study.

We achieved this by establishing a LC-MS/MS method for quantifying 15 compounds from a formula that consists of three herbs. The positive ESI with general source-dependent parameters was adopted for the ionization of the compounds. Three isomeric pairs were found: psoralen and isopsoralen, corylifolin and corylifolinin, and xanthotoxin and bergapten. The compounds in each pair exhibited the same mass spectrometric behavior and mass transition, but could not be differentiated by different ion channels. We found that methanol containing 0.2% formic acid was the most suitable eluent system. Under these conditions, all 15 compounds were resolved by retention time or differentiated by mass transition (MS/MS), allowing simultaneous quantification. These LC-MS/MS conditions were selective for all 15 components tested in the formula decoction and rat plasma without interference from other components in the formula extract and from normal endogenous plasma constituents.

The established LC-MS/MS method was partially validated in the pure water and rat plasma matrices against dynamic range, accuracy, precision, and the recovery and matrix effect for the pretreatment procedure of plasma samples prior to composition analysis application and PK test of BZ. The regression equations for all compounds in pure water and rat plasma all had good linearity (*r*>0.98) over the dynamic range from 1 or 5 to 100 or 1,000 ng/mL in both the pure water and the rat plasma matrixes. The recoveries for all compounds were consistent at 2.5, 5, 50, and 700 ng/mL and ranged from 83% to 107%. Thus, the developed method is sensitive, reliable, and suitable for both the composition and PK study. Compared with previously reported studies on *P. corylifolia* [36], *A. carmichaeli* [33], and *C. monnieri* [35], the advantages of this method were significantly demonstrated.

Using this method, the main compounds in the decoctions were determined. In D7, three alkaloids, aconitine, hypaconitine, and mesaconitine, the main toxic principles of *A. carmichaeli*, were not detected, which excludes their potential as the material basis for BZ. However, their transformation products, benzoylaconitine, benzoylmesaconine, and benzoylhypacoitine were present. The herb–herb interactions have been well documented in the preparation of TCM decoctions [37]–[39]. Thus, we also prepared and analyzed six other decoctions, D1 to D6. *C. monnieri* and *P. corylifolia* affect the dissolution of each other’s components, which were partially reversed by *A. carmichaeli*.

We identified the compounds that could be absorbed into the systemic circulation and reached certain levels by evaluating the PK properties of BZ in rats. As expected, only 5 out of 12 components were measureable in rat plasma for up to 10 h or 24 h after the single dose treatment (Figure 3). Furthermore, the PK parameters of the five compounds varied considerably, which might be the intrinsic characteristics of TCM formulas. Interestingly, psoralidin showed the lowest *C_max_* but the longest *T_1/2_*. Surprisingly, none of the compounds were obtained from *A. carmichaeli*. Together with the fact that no breast cancer-related reports on benzoylaconitine, benzoylmesaconine, benzoylhypacoitine were found, we assume that no material basis for BZ was derived from *A. carmichaeli*. However, the components of *A. carmichaeli* in BZ could not be underestimated because of its effect on the dissolution rate of the other two herbs. Recently, reports on the *in vitro* and *in vivo* anticancer effects of imperatorin [40] and osthole have been published [41], [42]. However, in the present study, their concentrations were below the detection limit. Therefore, the PK profiles provide five candidates for material basis.

We further identified the material basis by applying the network pharmacology strategy, which has recently been proven useful in TCM studies [23], [24]. Network pharmacology analysis revealed that psoralen-, psoralidin-, isopsoralen-, and bergapten-associated gene/protein form a network that shares nine key signaling pathway molecules/targets in carcinogenesis, progression, metastasis, and angiogenesis, such as TGF-β [43], COX-2 [44], EGFR [45], and so on. Xanthotoxin is outside the network and has no reported bioactivity so far. Thus, psoralen, psoralidin, isopsoralen, and bergapten in BZ may be the material basis for its beneficial effect in breast cancer. Xanthotoxin, however, could serve as a PK marker for BZ [46]. However, this study also showed some disadvantages. Beside that the PK results were based on the data obtained from rodents, the data for network pharmacological analysis are mainly obtained from *in vitro* studies. Furthermore, though the use of systems biology and related approaches are interesting approaches, they do not provide validated results. Thus, this study is an attempt to apply the network pharmacology theory to TCM and further systematic studies are needed in future.

In conclusion, the present study proposed a strategy to determine the material bases of TCM formulas by combining PK with network pharmacology. Using this strategy, a sensitive and credible LC-MS/MS method was firstly developed to determine 15 natural products simultaneously in a TCM formula that consists of three herbs. Among which, the PK profiles of 5 compounds were calculated and 4 compounds might be the material basis for BZ.
